# Genetic Analysis of *NDT80* Family Transcription Factors in *Candida albicans* Using New CRISPR-Cas9 Approaches

**DOI:** 10.1128/mSphere.00545-18

**Published:** 2018-11-21

**Authors:** Kyunghun Min, Amy Biermann, Deborah A. Hogan, James B. Konopka

**Affiliations:** a Department of Molecular Genetics and Microbiology, Stony Brook University, Stony Brook, New York, USA; b Department of Microbiology and Immunology, Geisel School of Medicine at Dartmouth, Hanover, New Hampshire, USA; Yonsei University

**Keywords:** *Candida albicans*, NDT80, REP1, RON1, hyphae, morphogenesis

## Abstract

Ndt80 family transcription factors are highly conserved in fungi, where they regulate diverse processes. The human fungal pathogen Candida albicans contains three genes (*NDT80*, *REP1*, and *RON1*) that encode proteins with similarity to Saccharomyces cerevisiae Ndt80, although the homology is restricted to the DNA binding domain. To better understand their role in virulence functions, we used clustered regularly interspaced short palindromic repeat/CRISPR-associated gene 9 (CRISPR/Cas9) to delete the three *NDT80*-family genes. An *ndt80*Δ mutant showed strong defects in forming hyphae in response to serum or *N*-acetylglucosamine (GlcNAc), which was linked to the ability of Ndt80 to regulate the expression of *RAS1*, an upstream regulator of hyphal signaling. Conversely, the *ndt80*Δ mutant formed hyphal cells on glycerol medium, indicating that Ndt80 is not required for hyphal growth under all conditions. In contrast to our previously published data, a *ron1*Δ single mutant could grow and form hyphae in response to GlcNAc. However, deleting *RON1* partially restored the ability of an *ndt80*Δ mutant to form hyphae in response to GlcNAc, indicating a link to GlcNAc signaling. *REP1* was required for growth on GlcNAc, as expected, but not for GlcNAc or serum to induce hyphae. The *ndt80Δ* mutant was defective in growing under stressful conditions, such as elevated temperature, but not the *ron1Δ* mutant or *rep1Δ* mutant. Quantitative assays did not reveal any significant differences in the fluconazole susceptibility of the NDT80-family mutants. Interestingly, double and triple mutant analysis did not identify significant genetic interactions for these *NDT80* family genes, indicating that they mainly function independently, in spite of their conserved DNA binding domain.

**IMPORTANCE** Transcription factors play key roles in regulating virulence of the human fungal pathogen C. albicans. In addition to regulating the expression of virulence factors, they also control the ability of C. albicans to switch to filamentous hyphal growth, which facilitates biofilm formation on medical devices and invasion into tissues. We therefore used new CRISPR/Cas9 methods to examine the effects of deleting three C. albicans genes (*NDT80*, *REP1*, and *RON1*) that encode transcription factors with similar DNA binding domains. Interestingly, double and triple mutant strains mostly showed the combined properties of the single mutants; there was only very limited evidence of synergistic interactions in regulating morphogenesis, stress resistance, and ability to metabolize different sugars. These results demonstrate that *NDT80*, *REP1*, and *RON1* have distinct functions in regulating C. albicans virulence functions.

## Abstract (precis)

Transcription factors play key roles in regulating virulence of the human fungal pathogen C. albicans. In addition to regulating the expression of virulence factors, they also control the ability of C. albicans to switch to filamentous hyphal growth, which facilitates biofilm formation on medical devices and invasion into tissues. We therefore used new CRISPR/Cas9 methods to examine the effects of deleting three C. albicans genes (*NDT80*, *REP1*, and *RON1*) that encode transcription factors with similar DNA binding domains. Interestingly, double and triple mutant strains mostly showed the combined properties of the single mutants; there was only very limited evidence of synergistic interactions in regulating morphogenesis, stress resistance, and ability to metabolize different sugars. These results demonstrate that *NDT80*, *REP1*, and *RON1* have distinct functions in regulating C. albicans virulence functions.

## INTRODUCTION

The human fungal pathogen Candida albicans commonly lives as a commensal on mucosal surfaces of most healthy humans, but it can initiate life-threatening systemic infections in those who are immunocompromised. Thus, C. albicans infections are the fourth most common type of nosocomial bloodstream infection (1). The attributable mortality rate is about 40% in spite of recent advances in antifungal therapy (2, 3), and the emergence of strains that are resistant to antifungal drugs is a further challenge to delivering effective therapy (4). Therefore, it is important to define the mechanisms of C. albicans pathogenesis in order to develop new therapies. One major factor that promotes C. albicans infections is its ability to grow in different morphologies ranging from budding cells to long chains of hyphal or pseudohyphal cells (5). The ability of C. albicans to grow as long filamentous hyphae is significant for its pathogenic potential as it promotes invasive growth into host tissues and biofilm formation (5, 6). Hyphal growth can be induced *in vitro* by a variety of environmental stimuli, including serum, alkaline pH, CO_2_, and *N*-acetylglucosamine (GlcNAc) (5, 7–9). Mutational analysis has identified a network of transcription factors (TFs) that are important for inducing the hyphal morphology. These TFs are also needed to induce a special set of genes during hyphal growth that encode factors needed for virulence, such as adhesin proteins and superoxide dismutase (5, 10–12).

**FIG 1:**
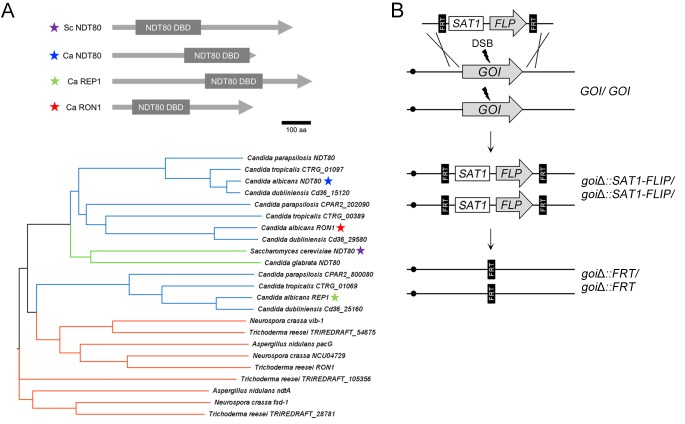
Strategy for multiple deletion of *NDT80*-family genes using transient CRISPR/Cas9 and the SAT1-FLP system. (A) In Candida albicans (Ca), there are three genes that encode proteins with similarity to S. cerevisiae (Sc) Ndt80. The amino acid sequence similarity is restricted to the DNA binding domain. The phylogenetic analysis of putative Ndt80 family proteins in *Ascomycota* indicates that Ndt80-like proteins can be assigned to two groups. One superbranch contains direct orthologs of S. cerevisiae Ndt80, such as Ca Ndt80 and Ca Ron1. Ca Rep1 clusters in the other superbranch, which features orthologs from *Pezizomycotina*. Colored stars indicate S. cerevisiae Ndt80 and C. albicans Ndt80 family proteins as follows. Color code: *Candida* CTG clade, blue; *Saccharomycetaceae*, green; *Pezizomycotina*, red. (B) The *CAS9* gene and the sgRNA were expressed transiently after transformation and were not integrated into the genome. The sgRNA targets Cas9 protein to produce a double-strand break (DSB) at a defined target sequence. The double-strand breaks can be repaired by homology-directed recombination with the SAT1-FLP cassette DNA fragment, which has homology on the ends to the target gene, to create a homozygous deletion of the gene of interest (GOI). The SAT1-FLP cassette confers nourseothricin (NAT) selection and marker recycling. Marker excision of the SAT1 gene is mediated by the maltose-inducible FLP recombinase, leaving a single FLP recombinase target (FRT) site in place of the each GOI.

One TF needed for hyphal growth in C. albicans is Ndt80 (10). The Ndt80 family is significant because it is highly conserved across a large group of fungal species (Fig. 1A). The number of Ndt80-like genes varies in different fungi, ranging from zero (Schizosaccharomyces pombe) and one (Saccharomyces cerevisiae) to six (Fusarium oxysporum strain HDV247) (13). They regulate diverse processes, including sexual development, filamentation, drug resistance, virulence, and the response to nutrient stress (13–16). One well-studied paralog is S. cerevisiae Ndt80, which is a key regulator of meiosis and sporulation (17, 18). In species that encode multiple Ndt80 homologs, genetic analyses indicate they can carry out distinct functions (13). In A. nidulans, one Ndt80-like protein (XprG) is a positive regulator that controls expression of an extracellular protease, mycotoxin production, and programmed cell death induced by carbon starvation (15), similarly to N. crassa VIB-1, which is required for expression of genes involved in heterokaryon incompatibility programmed cell death and, like XprG, is a positive regulator of extracellular protease production (14, 19). A. nidulans NdtA and N. crassa FSD-1, representing another type of Ndt80-related factor, have greater sequence similarity to S. cerevisiae Ndt80 and are required for sexual reproduction. Deletion of a third Ndt80-like gene in N. crassa (N. crassa U04729 [NCU04729]) had no effect on sexual reproduction or on any other phenotypes tested (14). Recently, functional analysis in Trichoderma reesei showed that one of Ndt80 homologs is a key activator of the GlcNAc gene cluster that is essential for GlcNAc catabolism (20).

There are three *NDT80* paralogs in C. albicans (*NDT80*, *RON1*, and *REP1*). *NDT80* has been shown to promote resistance to various stress conditions and to have a role in hyphal growth (21–23). We previously reported that a mutant lacking *RON1* displayed defects in growing on GlcNAc media and in inducing hyphae in response to GlcNAc (24), but we showed here that it is not required by the use of a new clustered regularly interspaced short palindromic repeat/CRISPR-associated gene 9 (CRISPR/Cas9) method for making gene deletions. The third paralog, *REP1*, was identified as a negative regulator of the expression of the drug efflux pump *MDR1* (25). *REP1* was also shown to be needed for growth on some sugars, such as GlcNAc and galactose (26), and is thought to act in part by recruiting Ngs1 to specific promoters to regulate histone acetylation (26). To compare the mutant phenotypes in the same strain background, and to determine whether the Ndt80-like TFs have overlapping functions, we constructed single, double, and triple mutant combinations. To circumvent the difficulties of creating homozygous mutations in the diploid C. albicans, we took advantage of recent developments in the application of CRISPR-Cas9 (27–31), including transient CRISPR-Cas9 and SAT1-Flipper (27, 32). The use of the SAT1-Flipper drug resistance marker enabled us to analyze mutant phenotypes in fully prototrophic strains such that their phenotypes were not affected by auxotrophies. Interestingly, the results showed that the functions of Ndt80-like TFs are largely independent despite the presence of the conserved DNA binding domain (DBD) in C. albicans.

## RESULTS

### Creation of gene deletion strains using transient CRISPR-Cas9 and SAT1-FLP systems.

To investigate the genetic interactions within the *NDT80* TF family, we constructed a set of mutants that contained homozygous deletion mutations for each TF, the three possible double mutants, and a triple mutant. The mutations were constructed using transient CRISPR-Cas9 and SAT1-FLP systems (Fig. 1B). In construction of single mutants, wild-type strain S. cerevisiae 5314 (SC5314) was transformed with Cas9 DNA, which is transiently expressed (27); a gene encoding a single guide RNA (sgRNA) to guide the CRISPR-Cas9 to a specific site in the target gene; and a repair template consisting of the recyclable SAT1-FLP cassette providing resistance to nourseothricin (NAT^r^) (32) flanked by regions of homology to the sequences surrounding the targeted open reading frame. We then created the double deletion mutants in the NAT^s^ versions of the single deletion mutants in which the NAT^r^ gene was excised. A triple deletion mutant of the *NDT80* TF family was generated in the *ron1*Δ *rep1*Δ NAT^s^ double mutant (Table 1). The frequency of creation of homozygous deletion mutants was similar to previous results for each round of gene deletions in the construction of the triple mutant (27).

**TABLE 1:** C. albicans strains used in this study

Strain	Short genotype	Parent	Genotype
SC5314	Wild-type strain		
KH1493	*ndt80*Δ	SC5314	*ndt80*Δ::*SAT1-FLIP*/*ndt80*Δ::*SAT1-FLIP*
KH1494	*rep1*Δ	SC5314	*rep1*Δ::*SAT1-FLIP*/*rep1*Δ::*SAT1-FLIP*
KH1495	*ron1*Δ	SC5314	*ron1*Δ::*SAT1-FLIP*/*ron1*Δ::*SAT1-FLIP*
KH1496	*rep1*Δ *ndt80*Δ	*rep1*Δ Nat^s^	*rep1*Δ::*FRT*/*rep1*Δ::*FRT ndt80*Δ::*SAT1-FLIP*/*ndt80*Δ::*SAT1-FLIP*
KH1497	*ron1*Δ *ndt80*Δ	*ron1*Δ Nat^s^	*ron1*Δ::*FRT*/*ron1*Δ::*FRT ndt80*Δ::*SAT1-FLIP*/*ndt80*Δ::*SAT1-FLIP*
KH1498	*ron1*Δ *rep1*Δ	*ron1*Δ Nat^s^	*ron1*Δ::*FRT*/*ron1*Δ::*FRT rep1*Δ::*SAT1-FLIP*/*rep1*Δ::*SAT1-FLIP*
KH1499	*ron1*Δ *rep1*Δ *ndt80*Δ	*ron1 rep1*Δ Nat^s^	*ron1*Δ::*FRT*/*ron1*Δ::*FRT rep1*Δ::*FRT*/*rep1*Δ::*FRT ndt80*Δ::*SAT1-FLIP*/*ndt80*Δ::*SAT1-FLIP*
KH1500	*ndt80*Δ + *NDT80*	*ndt80*Δ Nat^s^	*ndt80*Δ::*FRT*/*ndt80*Δ::*FRT rps1*::*NDT80*-CIp10-*SAT1*/*RPS1*
KH1501	*rep1*Δ + *REP1*	*rep1*Δ Nat^s^	*rep1*Δ::*FRT*/*rep1*Δ::*FRT rps1*::*REP1*-CIp10-*SAT1*/*RPS1*
KH1502	*ron1*Δ + *RON1*	*ron1*Δ Nat^s^	*ron1*Δ::*FRT*/*ron1*Δ::*FRT rps1*::*RON1*-CIp10-*SAT1*/*RPS1*
KH1503	*ndt80*Δ Nat^s^	*ndt80*Δ	*ndt80*Δ::*FRT*/*ndt80*Δ::*FRT*
KH1504	*rep1*Δ Nat^s^	*rep1*Δ	*rep1*Δ::*FRT*/*rep1*Δ::*FRT*
KH1505	*ron1*Δ Nat^s^	*ron1*Δ	*ron1*Δ::*FRT*/*ron1*Δ::*FRT*
KH1506	*ron1*Δ *rep1*Δ Nat^s^	*ron1*Δ *rep1*Δ	*ron1*Δ::*FRT*/*ron1*Δ::*FRT rep1*Δ::*FRT*/*rep1*Δ::*FRT*

### Growth on different carbon sources.

**FIG 2:**
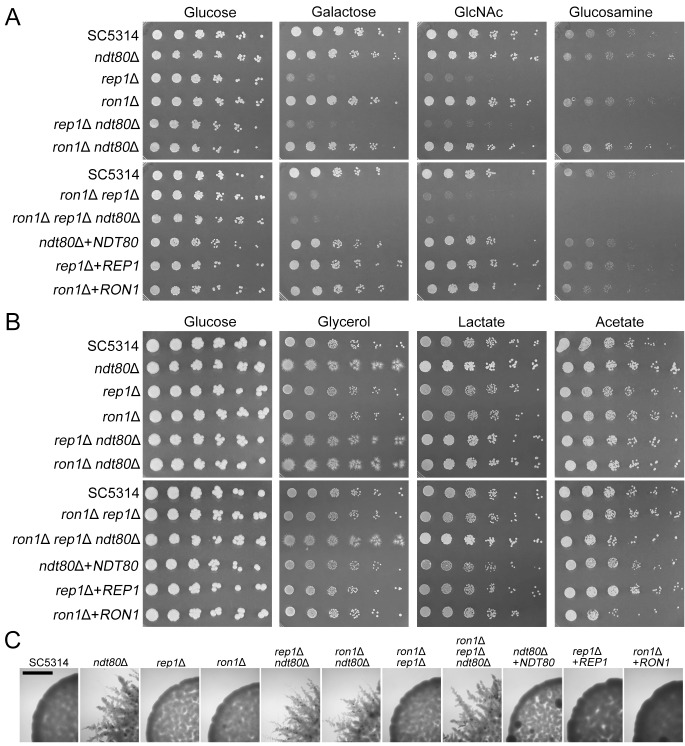
The *REP1* gene is needed for growth on galactose, GlcNAc, and glucosamine. Dilutions of cells were spotted onto minimal medium plates containing the indicated sugar. The genotype of the strain in each row is indicated on the left. The sugars were present at 50 mM, except for the plates containing glycerol, which was present at a higher concentration (300 mM) to promote better growth of the strains. The plates were incubated at 30°C for (A) 2 days or (B) 6 days and then photographed. The deletion mutants of *REP1* (strains *rep1*Δ, *rep1*Δ *ndt80*Δ, *ron1*Δ *rep1*Δ, and *ron1*Δ *rep1*Δ *ndt80*Δ) were specifically defective in growth on galactose, GlcNAc, and glucosamine. Note that the deletion mutants of *NDT80* (strains *ndt80*Δ, *rep1*Δ *ndt80*Δ, *ron1*Δ *ndt80*Δ, and *ron1*Δ *rep1*Δ *ndt80*Δ) grew slightly better on nonfermentative carbon sources (glycerol, lactate, and acetate). (C) The *ndt80*Δ cells grown on glycerol medium were distinct in that there were filamentous outgrowths of cells from the edges of the colonies. Scale bars indicate 1 mm. The strains used are listed in Table 1.

Cells lacking *NDT80* (C2_00140W) grew well on various carbon sources (Fig. 2A and B), similarly to the previous results (24). In fact, we found that the *ndt80*Δ mutant displayed slightly better growth on nonfermentable carbon sources (glycerol, lactate, and acetate) than the wild-type control. Interestingly, the *ndt80*Δ mutant showed filamentous hyphal cells emanating from colonies grown on the synthetic glycerol plates whereas other strains, including the wild-type SC5313 strain, formed smooth-edged colonies (Fig. 2C). This phenotype was surprising since Ndt80 has been reported to be required for hyphal growth in C. albicans (10, 12, 33, 34). These results indicate that Ndt80 can also act as a repressor of filamentous growth under some conditions. Cells lacking *REP1* (CR_04250W) showed a strong defect with respect to growth on the amino sugars GlcNAc and glucosamine. Rep1 was reported to regulate GlcNAc catabolic genes by recruiting Ngs1 to the promoters of the genes (26). However, the *rep1*Δ mutant was also unable to grow on galactose, indicating that it has broader roles in regulating metabolism (Fig. 2A). The deletion mutant lacking *RON1* (CR_04250W) did not exhibit a significant defect in growth on the different carbon sources. This contrasts with our previously published data (24) suggesting that mutant *ron1*Δ was defective in growth on GlcNAc and glucosamine. The reasons for these differences are unclear. However, we note that one difference is that the previous *ron1*Δ mutant was constructed in a different manner, including the use of auxotrophic markers for selection.

We next examined the set of double and triple homozygous mutants to determine if combining different mutations led to synergistic or compensatory growth phenotypes. However, none were detected (Fig. 2). All double and triple mutants lacking *REP1* (mutants *rep1*Δ *ndt80*Δ, *ron1*Δ *rep1*Δ, and *ron1*Δ *rep1*Δ *ndt80*Δ) did not grow well on galactose, GlcNAc, and glucosamine, similarly to the *rep1*Δ mutant. The multiple deletion mutants lacking *NDT80* (mutants *rep1*Δ *ndt80*Δ, *ron1*Δ *ndt80*Δ, and *ron1*Δ *rep1*Δ *ndt80*Δ) all showed slightly better growth on nonfermentable carbon sources such as was seen for the *ndt80*Δ single mutant.

### Hyphal morphogenesis.

**FIG 3:**
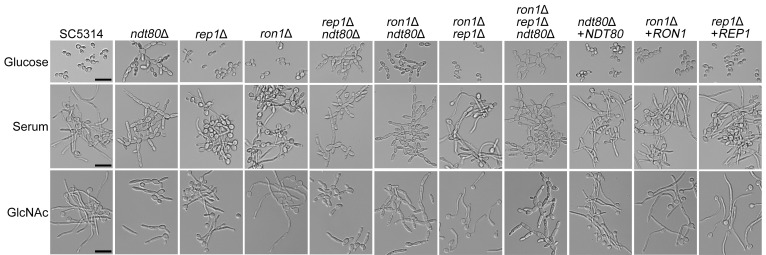
Deletion of *NDT80* caused defects in cell separation and hyphal growth. The strains indicated at the top were grown in the medium indicated on the left, and then cell morphology was assessed microscopically. Interestingly, the mutant strains lacking *NDT80* (strains *ndt80*Δ, *rep1*Δ *ndt80*Δ, *ron1*Δ *ndt80*Δ, and *ron1*Δ *rep1*Δ *ndt80*Δ) displayed a cell separation defect in minimal media containing 50 mM glucose, whereas the other mutants were similar to the wild-type control in that respect. Cells were also grown in liquid medium containing 15% serum or 50 mM GlcNAc to induce hyphal growth. The wild-type control and the *ron1*Δ mutant showed the formation of filamentous hyphal cells. In contrast, the *ndt80* mutant strains were defective, as they formed swollen, slightly elongated cells in the presence of the serum or GlcNAc. Since the *rep1*Δ mutant could not grow on GlcNAc, 5 mM glucose was added to the GlcNAc medium to support growth of the *rep1*Δ strains. The *rep1*Δ and *ron1*Δ *rep1*Δ mutants both grew well under these conditions and were induced *in vitro* to form hyphae by GlcNAc. Cells were incubated at 37°C for 4 h and then photographed. Scale bars indicate 20 µm.

Cell morphology was first examined after growth under conditions where the wild-type cells predominantly grew as budding cells (minimal glucose medium) (Fig. 3). In contrast, microscopic observation revealed that the *ndt80*Δ mutants formed connected chains of elongated cells. The multiple deletion mutants of *NDT80* (mutants *rep1*Δ *ndt80*Δ, *ron1*Δ *ndt80*Δ, and *ron1*Δ *rep1*Δ *ndt80*Δ) also showed a similar defect in morphology. To determine whether the mutant cells could form hyphae, they were induced in minimal medium containing either serum or GlcNAc (Fig. 3). As expected, the wild-type strain switched to forming filamentous cells under these conditions. Consistent with previous studies (34), the *ndt80*Δ mutant was strongly defective in hyphal growth as it instead formed short, elongated cells with a swollen appearance in the presence of the serum or GlcNAc.

Deletion of *REP1* did not affect hyphal induction in serum (Fig. 3). The *rep1Δ* mutant could not be induced in GlcNAc like the other strains, since it failed to grow on GlcNAc as the sole carbon source (Fig. 2A). On the basis of the inability of *rep1Δ* cells to grow on GlcNAc, it was suggested they might also be defective in inducing the transcriptional responses needed to form hyphae (26). In order to test this, the *rep1Δ* cells were induced in minimal GlcNAc medium supplemented with glucose to provide a source of energy. Although glucose can repress the induction of the GlcNAc transporter (35), the low concentration of glucose used in these experiments (5 mM) did not inhibit the hyphal induction by high levels of GlcNAc (50 mM). Interestingly, the *rep1*Δ mutant was stimulated efficiently by GlcNAc to form hyphae under those growth conditions (Fig. 3). Thus, our result demonstrated that Rep1 is not important for GlcNAc induction of hyphal morphogenesis.

Although we previously reported that Ron1 was defective in responding to GlcNAc to form hyphae in liquid medium (24), the *ron1*Δ mutant made with CRISPR/Cas9 approaches did not show any obvious defects in responding to GlcNAc or serum (Fig. 3). Interestingly, we also did not detect any synergistic defects in double and triple mutant combinations lacking *NDT80* family TFs.

**FIG 4:**
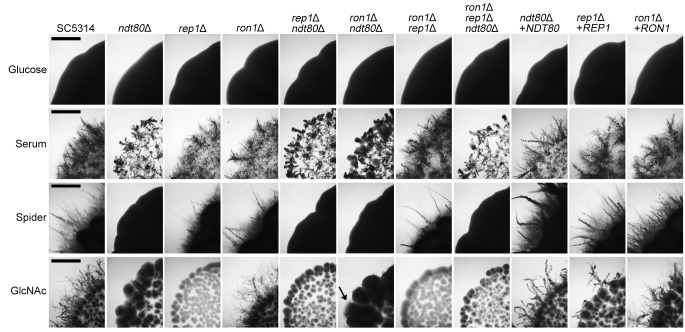
*NDT80* is needed for invasive hyphal growth into agar. The cells indicated at the top were spotted onto the agar plates listed on the left. Serum was present in the medium at 15% (vol/vol), and GlcNAc was present at 2.5 mM. The plates were incubated at 37°C and then photographed after 5 days to record the extent of invasive growth emanating from the edges of the colonies. The deletion mutants lacking *NDT80* (mutants *ndt80*Δ, *rep1*Δ *ndt80*Δ, *ron1*Δ *ndt80*Δ, and *ron1*Δ *rep1*Δ *ndt80*Δ) were strongly defective in invasive hyphal growth into agar media containing serum, Spider, or GlcNAc. Note that the *ron1*Δ *ndt80*Δ double mutant showed an improved ability to form hyphae on GlcNAc (arrow). The deletion mutants of *REP1* (mutants *rep1*Δ, *rep1*Δ *ndt80*Δ, *ron1*Δ *rep1*Δ, and *ron1*Δ *rep1*Δ *ndt80*Δ) grew poorly and were not detectably induced on GlcNAc. Scale bars indicate 1 mm.

The mutant cells were also tested for the ability to undergo invasive hyphal growth into agar in the presence of different types of media (Fig. 4). Control studies showed the expected results, i.e., that there were essentially no detectable hyphae emanating from the zone of growth for the fungal cells spotted onto minimal glucose medium. In contrast, the wild-type cells formed robust invasive hyphae 5 days after spotting of the cells onto the surface of serum, Spider, and GlcNAc agar plates. The *ndt80*Δ cells were strongly defective in invasive growth, as there were no hyphal outgrowths seen even after 5 days of incubation on serum and Spider media (Fig. 4). The *ndt80*Δ mutant showed only rare hyphal outgrowths on agar containing GlcNAc. The *rep1*Δ mutant was not defective in invasive growth into agar containing serum or Spider media. Although the *rep1Δ* mutant appeared to be strongly defective for invasion into GlcNAc medium, this was likely due to the poor growth of the *rep1*Δ mutant on GlcNAc as a sole carbon source (Fig. 2A). The *ron1*Δ mutant did not show an obvious defect in invasive hyphal growth on agar containing GlcNAc, in contrast to our previous result (24). However, the agar invasion studies revealed an interesting genetic interaction between *NDT80* and *RON1*. Deletion of *RON1* partially rescued the hyphal defect of the *ndt80*Δ mutant on GlcNAc (see the arrow in Fig. 4), indicating a role for *RON1* in GlcNAc regulation.

### Resistance to stress.

**FIG 5:**
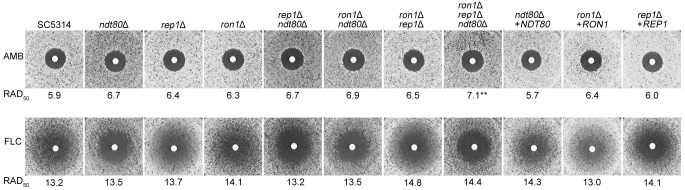
Sensitivity of mutants to antifungal drugs. The sensitivity to amphotericin B and fluconazole was determined by a disk diffusion assay in which cells were spread onto the surface of an RPMI 1640 medium plate and then filter discs containing 25 µg of the drugs were placed on the surface of the plate. The *ron1*Δ *rep1*Δ *ndt80*Δ triple mutant was slightly more sensitive to amphotericin B (AMB) than the wild-type SC5314 strain but was not more sensitive to fluconazole (FLC). None of the mutants showed significant differences in the zone of 50% growth inhibition (RAD_50_) in fluconazole compared to the wild-type strain. However, the deletion mutants lacking *NDT80* (mutants *ndt80*Δ, *rep1*Δ *ndt80*Δ, *ron1*Δ *ndt80*Δ, and *ron1*Δ *rep1*Δ *ndt80*Δ) did not show the trailing growth around the fluconazole disks that was seen for the other strains. The plates were incubated at 30°C for 48 h and then photographed. Image analysis software was used to measure the zone of growth inhibition to determine the average radius that corresponded to a 50% growth reduction (RAD_50_). Double asterisks show statistically significant differences (*P* < 0.01) from the wild-type strain based on a Student's *t* test.

We used a disk diffusion assay to measure the sensitivity of the mutant cells to the antifungal drugs amphotericin B and fluconazole. To quantitatively measure the drug sensitivity, we calculated the average radius in millimeters to the point where 50% growth reduction occurred (RAD_50_) using diskImageR image analysis software (see Materials and Methods). Statistical analysis showed that the single and double deletion mutants of *NDT80* family were similar to the wild type in amphotericin B sensitivity (Fig. 5). The triple deletion mutant (mutant *ron1*Δ *rep1*Δ *ndt80*Δ) showed a statistically significant increase in sensitivity to amphotericin B, but the magnitude of difference was minor. Although it has been reported that Ndt80 and Rep1 are involved in fluconazole susceptibility (21, 22, 25); surprisingly, our disk diffusion assays did not reveal any significant differences in the fluconazole susceptibility of the mutants compared to the wild type. However, we observed that all the deletion mutants lacking *NDT80* failed to show the trailing growth around the fluconazole disks that was seen for the wild-type strain (Fig. 5). Trailing growth (also called tolerance) is due to the slow growth of subpopulations of cells at antifungal drug concentrations above the MIC (36). Because these “tolerant” cells continue to divide in the presence of antifungals, they might contribute to the persistence and/or recurrence of fungal infections (37). Rosenberg demonstrated that fluconazole tolerance was clearly distinct from fluconazole susceptibility measured as MIC in C. albicans (37). Therefore, our result indicates that deletion of *NDT80* reduced drug tolerance of C. albicans without affecting the overall susceptibility to fluconazole.

**FIG 6:**
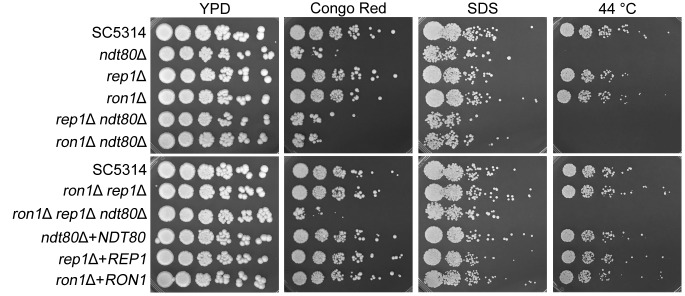
Sensitivity to cell wall stress and heat stress. The absence of *NDT80* resulted in increased sensitivity to Congo red and inhibition of growth at an elevated temperature (44°C). The deletion mutants of *NDT80* were also weakly sensitive to SDS. Dilutions of cells were spotted onto YPD plates containing the indicated chemicals. Congo red was present at 140 µM, and SDS was present at 0.06%. The plates were incubated at 30°C and then photographed after 3 days. To examine heat sensitivity, YPD cultures were incubated at 44°C and then photographed after 4 days.

The mutant cells were also tested for resistance to other stress conditions (Fig. 6). The *ndt80*Δ mutant grew poorly in Congo red, which binds and weakens the cell wall. The *ndt80*Δ mutant also showed sensitivity to the detergent SDS, which likely acts by disrupting the plasma membrane. These results indicate that deletion of *NDT80* resulted in defects in cell wall and plasma membrane integrity. A previous study reported that an *ndt80*Δ mutant showed slow growth at 42°C (23). Although we could not see a growth defect clearly at 42°C, the *ndt80*Δ mutant showed a very strong growth defect at 44°C (Fig. 6). Deletion of *REP1* or *RON1* did not appear to further enhance these phenotypes of the *ndt80*Δ mutant; the double and triple deletion mutants lacking *NDT80* (mutants *rep1*Δ *ndt80*Δ, *ron1*Δ *ndt80*Δ, and *ron1*Δ *rep1*Δ *ndt80*Δ) showed the same phenotypes as the *ndt80*Δ single mutant.

### Ndt80 regulates transcription of *RAS1*.

**FIG 7:**
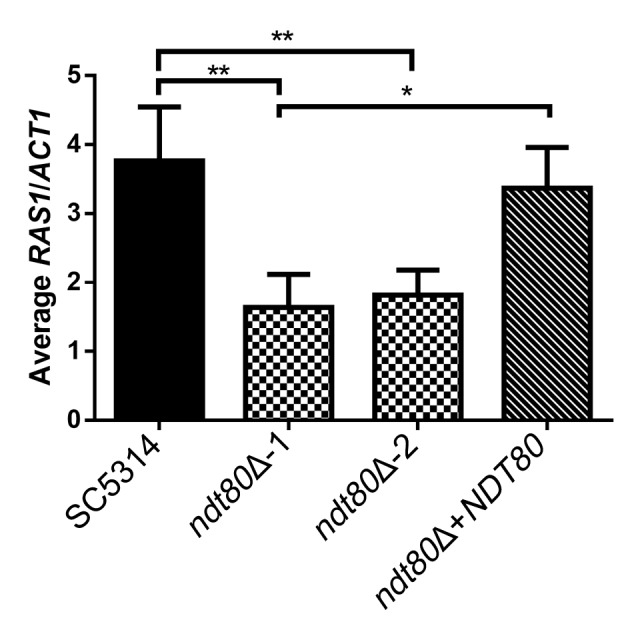
Ndt80 regulates expression of *RAS1*. Deletion of *NDT80* resulted in significantly reduced *RAS1* expression compared to wild-type strain SC5314. Complementation of an *ndt80*Δ mutant with *NDT80* restored *RAS1* expression to nearly wild-type levels. Cells were cultured under hypha-inducing conditions (YNBNP medium, pH 7.0, 37°C) for 1 h prior to RNA extraction and cDNA synthesis for qRT-PCR analysis. Values are shown as ratios of *RAS1* transcription to *ACT1* transcription. Asterisks show statistically significant differences (*, *P* < 0.05; **, *P* < 0.01) from the wild-type strain based on one-way analysis of variance (ANOVA) with multiple comparisons.

Given that Ndt80 has been found via chromatin immunoprecipitation (ChIP) to bind upstream of *RAS1* during biofilm formation (10) and that Ras1 is known to play a key role in the cAMP signaling that leads to hyphal growth, we investigated whether Ndt80 regulates *RAS1* expression. Using quantitative real-time PCR (qRT-PCR), we determined ratios of *RAS1* expression to *ACT1* expression in wild-type SC5314, *ndt80*Δ, and *NDT80*-complemented cells grown under hypha-inducing conditions. We observed that both *ndt80*Δ strains tested had significantly reduced *RAS1*/*ACT1* ratios compared to SC5314 (Fig. 7). Complementing *NDT80* back into an *ndt80*Δ strain restored *RAS1* expression to nearly wild-type levels (Fig. 7). Taken together, these results suggest that Ndt80 was a positive regulator of *RAS1* expression under the conditions tested.

## DISCUSSION

### Use of a recyclable marker system for CRISPR-Cas9 analysis of the *NDT80* family.

We further improved the previously reported transient CRISPR-Cas9 system (27) by using a SAT1-FLP system (32) for marker recycling. In this transient CRISPR system, Cas9 and sgRNA are transiently expressed from linear DNA fragments that are not stable in the cells, thereby avoiding integration of CRISPR/Cas9 into the C. albicans genome and the potential problems that could arise from long-term expression of this enzyme (27). The incorporation of a fusion PCR method for generating new sgRNAs streamlined the process of making gene deletions. However, the original system was not adequate to produce multiple deletion mutants since it did not use recyclable markers (27). Huang and Mitchell solved this problem by surrounding the selectable markers with direct repeats, enabling CRISPR-directed marker recycling. In their approach, two auxotrophic marker genes could be used to sequentially delete three or more genes in the same strain (30). Another approach for recycling selectable markers was used by Nguyen et al. in which CRISPR-Cas9 and a selectable marker were integrated in tandem into the genome and were then excised after confirming that the expected genome editing event had occurred (31). Although this method enables rapid gene editing, the initial stable genome integration results in constitutive expression of CRISPR-Cas9 system that could induce off-target mutations in the genome. In the present study, we therefore combined the use of transient CRISPR-Cas9 and a recyclable SAT1-FLP marker for selection. This approach allowed us to construct single, double, and triple mutant combinations of the three *NDT80* family genes in a wild-type strain background with no concerns about interference due to auxotrophic markers used for selection and the fact that transient CRISPR/Cas9 system does not generate a permanent disruption of a genomic locus.

### Roles of Ndt80 family transcription factors in filamentous hyphal growth.

Using the new CRISPR/Cas9 approaches, we analyzed the effects of deleting the three *NDT80*-family genes in the same strain background without the use of auxotrophic selection markers. The *ndt80*Δ mutant was defective in forming hyphae in response to the strong inducers serum and GlcNAc, as expected. Interestingly, we found that Ndt80 regulates the expression of *RAS1*, which is a key upstream component of the cAMP signaling pathway that is needed for hyphal growth (Fig. 7). These results are consistent with ChIP sequencing (ChIP-Seq) data, which have shown that Ndt80 binds to the promoter region of the *RAS1* gene (12). However, the *ndt80Δ* mutant appears to have a stronger hyphal defect than a *ras1*Δ mutant (38, 39) indicating that Ndt80 must also regulate other aspects of hyphal growth. In addition, the *ndt80*Δ mutant showed abnormal cell morphology under budding conditions, which is not seen for *ras1*Δ mutants (38, 39). Interestingly, Ndt80 was not essential for filamentous growth under all conditions as *ndt80Δ* cells grown on glycerol medium instead of glucose formed hyphae (Fig. 2B and C). This contrasting phenotype implies that the requirement for Ndt80 in filamentous growth changes depending on the environmental condition.

In this study, deletion of *RON1* did not reveal significant phenotypes, in contrast to our previous study of a *ron1*Δ mutant made with a different approach that indicated a role for *RON1* in regulating GlcNAc signaling (24). However, the *ndt80*Δ *ron1*Δ double mutant had an improved ability to form hyphae on GlcNAc medium, which is consistent with a role for *RON1* in GlcNAc regulation. This phenotype suggests that *RON1* can act as a negative regulator of filamentous growth response to GlcNAc in the absence of *NDT80*. ChIP-Seq performed by Nocedal et al. showed that Ron1 binds to many fewer genomic regions but that all those sites are also bound by Ndt80 (12). This suggests that Ron1 may influence expression of a subset of genes regulated by Ndt80.

Our results indicated that Rep1 was not needed for hyphal induction when cells were stimulated by GlcNAc (Fig. 3). This contrasts with a suggestion from the previous study by Su et al. (26). They predicted that Rep1 could be involved in stimulating hyphal growth because it is needed to induce GlcNAc catabolic genes by recruiting the histone acetyltransferase, Ngs1, to the promoter region of these GlcNAc genes (26). However, when the *rep1*Δ mutant was supplied with an alternative sugar to promote growth, it could be induced to form hyphae in response to GlcNAc (Fig. 3). This is consistent with the previous finding that GlcNAc induction of hyphal morphogenesis in C. albicans is not dependent on its metabolism (40).

### Roles of *NDT80* family transcription factors in response to drugs and stress.

Previous studies reported that the *ndt80*Δ mutant was sensitive to fluconazole whereas the *rep1*Δ mutant was resistant to fluconazole (22, 25). The authors of those studies used spot assays, which do not quantify the changes in drug sensitivity. To compare fluconazole sensitivities in a quantitative manner, we used disk diffusion assays, which showed that three different deletion mutants lacking the *NDT80* family genes were not significantly different from the wild-type strain (Fig. 5). Instead, we found that the *ndt80*Δ mutant was distinct in that it did not show the typical trailing growth that is seen with the wild-type strain. Rosenberg et al. demonstrated that this type of drug tolerance is clearly distinct from susceptibility/resistance measured as MIC or halo size (37). We also found that the *ndt80*Δ mutant was susceptible to Congo red, SDS, and high temperature (Fig. 6). These phenotypes suggest that Ndt80 has multiple functions in stress response under various conditions.

### Limited genetic interactions among the members of the *NDT80* family.

We hypothesized that the three *NDT80* family genes might display strong genetic interactions due to the similarity of their DBDs. Surprisingly, in spite of the fact that a previous study indicated that Ron1 binds to a subset of Ndt80 binding sites in the genome, we observed only a weak genetic interaction between *ndt80Δ* and *ron1*Δ for GlcNAc-induced hyphal growth and did not detect other evidence for genetic interactions. Furthermore, deletion of *REP1* did not alter the hyphal defect of the *ndt80*Δ mutant or show a genetic interaction with *ron1*Δ. Similarly, deletion of *RON1* or *NDT80* did not change the growth defect of the *rep1*Δ mutant on galactose, glucosamine, or GlcNAc, which is consistent with the fact that Ndt80 and Ron1 did not bind to the promoters of GlcNAc catabolic genes (12) that are reported to be bound by Rep1 (26). These results suggest that the three *NDT80* family TFs are functionally independent and that their regulatory networks do not affect each other. A further sign of this is that Nocedal et al. did not detect the binding of Ndt80 or Ron1 to each other’s promoter regions or to the promoter region of *REP1* (12). Consistent with this, even the *ron1*Δ *rep1*Δ *ndt80*Δ triple mutant displayed a phenotype similar to the combined phenotypes of the corresponding single mutants.

The failure to detect genetic interactions for the *NDT80* family is in sharp contrast to the results of analysis of other genetic networks, such as the transcription factors involved in biofilm formation (Bcr1, Brg1, Efg1, Ndt80, Rob1, and Tec1) (33). In the latter case, many double mutants showed synergistic defects and were severely defective in biofilm formation. The biofilm network showed properties of a highly interdependent network that is susceptible to genetic perturbation (10, 33). It is interesting that Ndt80 is highly interconnected with other transcription factors in the biofilm network whereas it is largely independent of the other *NDT80* family transcription factors in spite of their conserved DBD. The roles for the three *NDT80*-family genes in C. albicans therefore appear to be similar to the function of the multiple *NDT80*-family genes found in filamentous fungi. The *NDT80* family TFs in filamentous fungi fall into two main groups; one group has functions in sexual development, and the other has various functions in metabolism, including GlcNAc catabolism (13–15, 19). Taken together, these results help us to understand the role of the highly conserved NDT80 family of transcription factors in fungi.

## MATERIALS AND METHODS

### Strains and culture conditions.

The genotypes of the C. albicans strains that were used are described in Table 1. All strains were stored in 15% glycerol stocks at −80°C. All C. albicans strains were streaked from −80°C onto YPD (1% yeast extract, 2% peptone, 2% glucose) plates every week and incubated at 30°C. C. albicans transformants were selected on YPD plus 200 µg/ml nourseothricin (NAT; Werner BioAgents) for nourseothricin-resistant (Nat^r^) isolates. To obtain nourseothricin-sensitive (Nat^s^) derivatives, transformants were grown overnight in maltose medium (1% yeast extract, 2% peptone, 2% maltose). Cells were then spread on YPD plates containing 25 µg/ml of NAT and incubated for 2 days at 30°C. Nat^s^ colonies were screened by their colony size, which was smaller than that of their Nat^r^ parental strains.

Fungal strains were tested for growth on different sugars by spotting dilutions of cells on synthetic agar medium containing 0.67% yeast nitrogen base with ammonium sulfate and the indicated carbon source. Fungal strains were also tested for resistance to different stress conditions by spotting dilutions of cells on YPD agar medium containing 140 µM Congo red or 0.06% SDS or by incubation at 44°C. Strains were grown overnight in YPD with shaking and adjusted to 2 × 10^7^ cells/ml, and then 5-fold dilutions of cells were prepared. A 3-µl aliquot of each dilution was then spotted onto the indicated type of plate, and the plates were incubated at 30°C or 44°C as indicated.

The ability to form hyphae in liquid media was analyzed with cells grown overnight at 30°C in YPD. The cells were then diluted to 2 × 10^6^ cells/ml into synthetic medium containing 50 mM glucose. After 3 h of incubation at 30°C, hyphal growth was induced by a shift in temperature to 37°C in combination with 15% bovine serum. For *N*-acetylglucosamine (GlcNAc) induction, the cells were diluted into synthetic medium containing 50 mM GlcNAc and incubated at 37°C. Glucose (5 mM) was added to the GlcNAc medium to support growth of the *rep1*Δ strains.

Invasive hyphal morphogenesis was analyzed by spotting 3 µl of serial dilutions of cells on an agar plate with the indicated type of medium and then incubating at 37°C. Cells were induced on synthetic medium containing 2.5 mM GlcNAc, agar medium containing 15% bovine serum, or Spider medium. The morphology of the cells at the edge of the zone of growth was photographed to record the extent of invasive hyphal growth into the agar.

### Assaying susceptibility to antifungal drugs with disk diffusion assays.

C. albicans cells were grown overnight in YPD, and then 2.5 × 10^5^ cells were spread onto 20-ml RPMI 1640 agar plates. Stock solutions of amphotericin B (Sigma-Aldrich Corp.) and fluconazole (Sigma-Aldrich Corp.) were prepared at 10 mg/ml in dimethyl sulfoxide (DMSO). A 2.5-µl volume of the stock solution (25 µg) was spotted onto 6-mm-diameter paper disks and allowed to dry in the dark. A single paper disk was placed in the center of each plate, and then the plates were incubated at 30°C for 48 h and each plate was photographed individually. Analysis of the disk diffusion assay was done using the diskImageR package from the R library. The diskImageR analysis measured pixel intensity corresponding to cell density and calculated the average radius in millimeters to the point where 50% growth reduction occurred (RAD_50_) (41).

### Macroscopic and microscopic analysis of cells.

Cells were allowed to grow on solid agar media for the indicated number of days and then photographed. Unless otherwise noted, all spot assays were completed as at least three independent replicates, and a representative data set is shown. To record the extent of invasive hyphal growth into the agar, the cells at the edge of the zone of growth were photographed using an Olympus BH2 microscope equipped with a 4× objective and a Zeiss AxioCam digital camera. To image the morphology of cells under hypha-inducing conditions, the cells in liquid medium were concentrated by centrifugation and then photographed using differential interference contrast (DIC) optics. Photographic images were captured using a Zeiss Axio Observer 7 inverted microscope equipped with a 100× objective and a Zeiss AxioCam 702 digital camera.

### DNA and plasmids.

The single, double, and triple mutant strains of *NDT80* family were created using transient expression of CRISPR-Cas9 to facilitate the homozygous deletion of a target gene. The methods were performed essentially as described previously (27). We used 20-bp target sequences of the sgRNA, as reported previously by Vyas et al. (28), to delete *NDT80* (target sequence GCATGCCCGTATTGATAGA), *REP1* (TGTAGTGTAGCCATACTCGC), and *RON1* (TCTCCCCACTTAAAGCAGCT). Gene deletion PCR constructs were synthesized using plasmid pGR-NAT as the template. The pGR-NAT plasmid has a full cassette of *SAT1 flipper* (32). The primers were designed to include 60 to 80 bases of homology to the sequences upstream or downstream from the target gene. PCR was conducted with *Ex Taq* in accordance with the manufacturer’s instructions (TaKaRa Bio, Inc.).

The complemented strains of the *NDT80* family were constructed by integrating a copy of the wild-type gene sequence into the genome. The *NDT80*, *REP1*, and *RON1* plasmids were constructed by PCR amplification of genomic DNA from 1 kb upstream of the start codon and 0.5 kb downstream of the stop codon of the *NDT80*, *REP1*, and *RON1* genes, respectively. PCR was conducted with Phusion in accordance with the instructions of the manufacturer (New England Biolabs, Inc.). The primers were designed to include 20 bases with homology to CIp10-SAT1, a SAT1-marked version of plasmid CIp10 (42, 43). The DNA fragments were then inserted at EcoRI restriction site of the CIp10-SAT1 plasmid using Gibson assembly master mix (New England Biolabs, Inc.). The constructed plasmids were linearized by the use of StuI and integrated into the *RPS1* locus of the fungal strains.

### Fungal transformation.

PCR products and linearized plasmids for transformation were purified and concentrated by the use of a QIAquick PCR purification kit (Qiagen N.V.). Electroporation was used to introduce the DNA into the cells in order to obtain optimal transformation efficiency. The electrocompetent fungal cells were prepared following a previously described method (44). A cell suspension (40 µl) was added to aliquoted DNA, placed in electroporation cuvettes, and electroporated on a Bio-Rad Gene Pulser at 1.5 kV. One milliliter of 0.5× YPD containing 1 M sorbitol was added immediately to the cuvette, and then the cell mixture was incubated for 3 h at 30°C before plating onto selective media was performed. Nat^r^ transformants were selected, and PCR genotyping of the transformants verified that both copies of the target gene had been deleted. Marker excision was mediated by the maltose-inducible FLP recombinase, and NAT^s^ cells were screened as described above (32).

### qRT-PCR.

Fungal cells were grown overnight in YPD, washed once with phosphate buffer (pH 7.0), and resuspended in phosphate buffer. Approximately 4.5 × 10^6^ cells were inoculated in 5 ml YNBNP medium (0.67% YNB, 0.2% glucose, 25 mM phosphate buffer, 5 mM GlcNAc) at pH 7.0 and incubated at 37°C for 1 h. RNA was extracted using a MasterPure yeast RNA purification kit (Epicentre). DNA contamination was removed by the use of a Turbo DNA-free kit (Invitrogen). cDNA was synthesized from 500 ng DNase-treated RNA using a RevertAid H Minus First Strand cDNA synthesis kit (Thermo Scientific), following the manufacturer’s instructions for the random hexamer primer (IDT) and GC rich template. qRT-PCR was performed on a CFX96 real-time system (Bio-Rad), using SsoFast Evergreen Supermix (Bio-Rad) with the indicated primers to detect *ACT1* (forward primer, 5′-ACTACCATGTTCCCAGGTATTG-3′; reverse primer, 5′-CCACCAATCCAGACAGAGTATT-3′) and *RAS1* (forward primer, 5′-TATCAAGATGGATTAGCATTCG-3′; reverse primer, 5′-ATATTGGTCTTGACCTTGTTG-3′). Thermocycler conditions were as follows: 95°C for 30 s followed by 40 cycles of 95°C for 5 s, 65°C for 3 s, and 95°C for 5 s. *RAS1* transcripts were normalized to *ACT1* transcripts.
